# Sensitization-Associated Symptoms and Neuropathic-like Features in Patients with Cervical Dystonia and Pain

**DOI:** 10.3390/jcm13072134

**Published:** 2024-04-07

**Authors:** Diego de-la-Hoz-López, María L. Cuadrado, Eva López-Valdés, Rocío García-Ramos, Fernando Alonso-Frech, Ana Fernández-Revuelta, César Fernández-de-las-Peñas, Víctor Gómez-Mayordomo

**Affiliations:** 1 Department of Medicine, School of Medicine, Universidad Complutense, 28040 Madrid, Spainmlcuadrado@med.ucm.es (M.L.C.); evalopezvaldes@yahoo.es (E.L.-V.); garciaramosg@gmail.com (R.G.-R.); f.frech@yahoo.es (F.A.-F.); ana93fr@gmail.com (A.F.-R.); 2 Department of Neurology, Hospital Clínico San Carlos, 28040 Madrid, Spain; 3 Department of Physical Therapy, Occupational Therapy, Physical Medicine and Rehabilitation, Universidad Rey Juan Carlos, 28922 Madrid, Spain; 4 Synaptia Institute of Neurosciencies, Hospital Universitario Vithas Madrid La Milagrosa, 28010 Madrid, Spain; vicmayordomo@gmail.com

**Keywords:** cervical dystonia, sensitization, neuropathic, pressure pain threshold, pain, anxiety, depression, sleep, phenotype

## Abstract

**Background:** This exploratory study evaluated the presence of sensitization-associated and neuropathic-like symptoms and identified their association with pressure sensitivity, pain, and disability in patients with cervical dystonia (CD). **Methods:** Thirty-one patients with CD (74.2% women, age: 61.2 years, SD 10.1) participated. Data collected included clinical variables, the Toronto Western Spasmodic Torticollis Rating Scale (TWSTRS), the Central Sensitization Inventory (CSI), the Self-administered Leeds Assessment of Neuropathic Symptoms and Signs (S-LANSS), the Hospital Anxiety and Depression Scale (HADS) and the Pittsburgh Sleep Quality Index (PSQI), as well as widespread pressure pain thresholds (PPTs). **Results:** Patients with CD with pain (*n* = 20, 64.5%) showed higher scores on the TWSTRS disability subscale and the CSI (*p* < 0.001), and lower PPTs (*p* < 0.05). Fifteen patients (15/31, 48%) showed sensitization-associated symptoms (CSI ≥ 40), whereas five of the patients with pain (5/20, 25%) exhibited neuropathic-like symptoms (S-LANSS ≥ 12). The CSI and S-LANSS were positively associated with the TWSTRS, HADS-A and HADS-D, and negatively associated with PPTs. HADS-D and S-LANSS explained 72.5% of the variance of the CSI (r^2^: 0.725), whereas CSI explained 42.3% of the variance of the S-LANSS (r^2^: 0.423). **Conclusions:** Pain is an important source of disability in CD, and may be a consequence of different mechanisms, including sensitization.

## 1. Introduction

Dystonia is a clinical syndrome characterized by involuntary muscle contractions that lead to sustained or intermittent twisting movements or abnormal postures [1]. Dystonic movements typically exhibit a pattern, are exacerbated due to voluntary actions, and often involve overflow muscle activation [2]. Another common feature is the presence of sensory tricks, which are voluntary maneuvers or stimuli such as a light touch to a particular area of the skin that temporarily relieve the symptoms of dystonia [1,2].

Cervical dystonia (CD) is a specific type of focal dystonia affecting the muscles of the neck. CD usually develops progressively over several months or years and then remains stable, with spontaneous remissions occurring in less than 10% of the cases [3]. A spreading pattern of dystonic movements to regions of the body other than the cervical spine can be observed in 8% of patients with CD [4]. To date, there is no curative or disease-modifying treatment for CD. 

Pain is a frequent non-motor symptom in CD, which can reach a peak prevalence of 90% [5]. Thus, pain is one of the most disabling symptoms of CD and is one of the main reasons why patients seek treatment [6]. The pathophysiology of pain in CD is not completely understood. Traditionally, it has been attributed to sustained muscle contractions and the sudden twisting motion of the neck. However, no correlation between the presence of pain and the degree of muscle contraction has been found [7]. In fact, evidence supports that central mechanisms (e.g., altered nociceptive processing, dysfunction of descending pain inhibitory pathways, and/or structural and network changes in the basal ganglia and cortex) may contribute to pain in CD [8]. Interestingly, sensory tricks have been found to be more frequent in patients with CD and pain than in those without pain [9].

The presence of central mechanisms, i.e., central sensitization, is common in most chronic pain conditions [10]. Central sensitization is the underlying concept for defining nociplastic pain. The International Association for the Study of Pain (IASP) has defined nociplastic pain as “pain that arises from altered nociception despite no clear evidence of actual or threatened tissue damage causing the activation of peripheral nociceptors or evidence for disease or lesion of the somatosensory system causing the pain” [11]. Nociplastic pain is not just associated with augmented pain responses but also with other symptoms such as psychological disturbances, e.g., anxiety or depression, and sleep problems [12]. 

Directly measuring central sensitization remains challenging due to the absence of a gold standard for distinguishing between normal and heightened pain responses in individual patients. In such a scenario, quantitative sensory tests, e.g., pressure or thermal pain thresholds, and/or self-reported questionnaires have been used for the evaluation of sensitization-associated symptoms. In a previous study, nine CD patients demonstrated lower pressure pain thresholds in both cervical and masticatory muscles when compared to controls [13]. Yet, no prior research has taken a comprehensive approach to explore the presence of sensitization-associated symptoms in CD, incorporating both quantitative sensory testing and self-reported questionnaires. The aims of this study were to evaluate the presence of sensitization-associated and neuropathic-like symptoms and to identify their association with pressure pain sensitivity, pain, and disability in patients with CD. 

## 2. Methods

### 2.1. Participants

Between November 2022 and October 2023, an exploratory, cross-sectional, observational study was conducted at the Movement Disorders Unit of the Neurology Department of Hospital Clínico San Carlos (Madrid, Spain). We specifically enrolled consecutive patients diagnosed with CD who met the following inclusion criteria: (1) aged between 18 and 80 years old; (2) a diagnosis of isolated idiopathic CD according to the Movement Disorders Society criteria [1,14], and (3) a minimum of 12 weeks since the last administration of botulinum toxin infiltration for the treatment of CD. Patients were excluded when they presented any of the following exclusion criteria: (1) identification of secondary CD or clinical evidence suggesting a secondary cause (such as the presence of structural lesions or fixed postures from onset and/or other positive signs on examination consistent with a functional movement disorder); (2) having received physical therapy sessions within the last month, including acupuncture or dry needling; (3) having received local injections within the past three months; (4) any change in pharmacological treatment for dystonia in the previous month; (5) a history of surgery, whiplash, or cervical radiculopathy; (6) concomitant severe systemic disease; (7) any comorbid chronic pain condition; (8) difficulty understanding and/or complying with informed consent requirements or accurately documenting symptoms, and (9) refusal to participate. 

The study was approved by the Clinical Research Ethics Committee of Hospital Clínico San Carlos (code 22/255-E). All participants received detailed written and verbal information about the study and confirmed their consent by signing an informed consent form.

### 2.2. Demographic and Clinical Variables

The demographic and clinical variables included: age, sex, time (years) of evolution of CD, the co-occurrence of head tremor, sensory tricks, the presence of pain, and drugs used for pain. The prevailing pattern of CD observed in each patient was also determined: torticollis (horizontal head rotation), laterocollis (lateral deviation), retrocollis (head extension), or anterocollis (head flexion) [15]. 

In those patients reporting pain, we collected the distribution and side of the pain, the intensity of the pain in the previous week on a numerical pain rating scale (NPRS, 0: no pain, 10: the worst pain imaginable), and the temporal pattern of the pain (continuous or intermittent). 

The Toronto Western Spasmodic Torticollis Rating Scale (TWSTRS) was used to assess the severity of dystonia and its related consequences, including disability and pain. TWSTRS scores range from 0 to 85, comprising three subscales: (1) dystonia severity, which focuses on the motor manifestations of CD (0–35); (2) disability (0–30), and (3) pain (0–20). This scale has demonstrated good interobserver reliability, convergent validity, and sensitivity to changes [16]. 

### 2.3. Sensitization-Associated Symptoms

The Central Sensitization Inventory (CSI) was used to detect symptoms associated with sensitization. This self-reported questionnaire evaluates the presence of 25 different symptoms that reflect various facets of sensitization [17]. Each item is rated on a 5-point Likert scale leading to a score ranging from 0 to 100, where ≥40 points suggest the presence of central sensitization [18]. The CSI has shown proper psychometric strength for evaluating sensitization-associated symptoms in patients with persistent pain [19,20].

### 2.4. Neuropathic Pain Assessment

The self-administered Leeds Assessment of Neuropathic Symptoms and Signs (S-LANSS) scale was used to identify pain with neuropathic features. This scale consists of seven items, with five focusing on the patient’s pain experience and two assessing clinical signs; these two items involve a self-examination by the patient to identify allodynia and hyperalgesia. All items prompt dichotomous responses (yes/no). The total score spans from 0 to 24 points, with a score of 12 or above suggesting the presence of neuropathic pain. The S-LANSS has shown good sensitivity, internal consistency, and validity [21].

### 2.5. Psychological and Sleep Variables 

The Hospital Anxiety and Depression Scale (HADS) was used to evaluate the presence of anxiety and depressive symptoms [22]. The HADS consists of two subscales, the first assessing anxiety (HADS-A, seven-items, 0–21 points) and the second assessing depression (HADS-D, seven items, 0–21 points). Higher scores correspond to higher levels of anxiety and/or depression. We considered the recommended cut-off scores for the Spanish population, indicating the presence of anxiety (HADS-A ≥ 12 points) and/or depression (HADS-D ≥ 10 points) [23]. 

The Pittsburgh Sleep Quality Index (PSQI) was used to evaluate sleep quality [24]. It consists of 19 questions assessing different aspects of sleep during the previous month. The score ranges from 0 to 21 points, where higher scores indicate worse sleep quality, and a score of 8 points or above indicates poor sleep [24]. 

### 2.6. Pressure Pain Thresholds

For evaluating widespread pressure pain sensitivity, pressure pain thresholds (PPTs) were measured bilaterally over the C5-C6 zygapophyseal joint, the upper trapezius muscle, the second interossei space, and the tibialis anterior muscle. PPTs refer to the minimum pressure required to transition from a sensation of pressure to a sensation of pain. For PPT measurement, a pressure algometer was used, equipped with a rubber tip of 1 cm in diameter (Pain Diagnosis and Treatment Inc., Great Neck, NY, USA). The algometer was calibrated before each data record. Pressure was applied perpendicular to each point at a speed of approximately 1 kg/cm^2^/s. To prevent temporal summation, which can arise from mechanical stimulation within the range of 0.16 Hz to 1 Hz, we incorporated a 30-s resting period between each trial [25]. The mean of three trials on each point was calculated and used in the main analyses.

No analgesics were allowed 24 h prior to evaluation and collection of all data.

### 2.7. Sample Size Determination

Although this was an exploratory study, we assumed that an adequate sample size for prediction models should comprise around 10 subjects per potential predictor variable, with no more than three predictors per model [26]. Accordingly, for three predictor variables a minimum of 30 participants was set. 

### 2.8. Statistical Analysis

Statistical analyses were performed using the IBM SPSS Statistics v.28 for Windows. The mean and standard deviation (SD) are presented for quantitative data, whereas absolute values and percentages are presented for categorical variables. The Kolmogorov–Smirnov test was used to test whether data followed a normal distribution. Paired Student t-tests were used to evaluate side-to-side differences for PPTs. Since no differences were found, the mean of both sides on each assessed area was used in the main analyses.

Differences in age, time of evolution of CD, TWSTRS, CSI, S-LANSS, HADS-A, HADS-D, PSQI and PPTs between patients with and without pain were assessed with independent Student t-tests; sex distributions and the presence of head tremors and sensory tricks in the two groups were compared with the chi-square test. In addition, multiple hierarchical regression analyses were used to determine the variables explaining the variance of CSI or S-LANSS. First, correlations between predictors and the dependent variables were assessed using a Pearson correlation (r) matrix. The Pearson coefficient was also used to assess multicollinearity between the variables (r > 0.8). All statistically significant variables associated with CSI or S-LANSS were included into multiple hierarchical regression analyses to identify independent variables that contributed significantly to the variance of each variable, except those showing multicollinearity. The significance criterion of the critical F value for entry into the regression equation was set at *p* < 0.05. Changes in adjusted R2 were reported after each step of the model to determine the association of additional variables.

## 3. Results

A total of 46 patients attending the Movement Disorders Unit of the Neurology Department of Hospital Clínico San Carlos were screened for eligibility criteria. Of these, 15 (32.6%) were not eligible for several reasons: four patients were diagnosed with segmental dystonia also affecting craniofacial or upper limb muscles, three were identified with functional dystonia, one had a tardive dystonia, one was suffering from cervical radiculopathy, and six declined to participate in the study. Finally, 31 patients with CD were included, with 74.2% being women and a mean age of 61.2 years (SD: 10.1 years). The prevalence of pain in this sample was 64.5% (20 patients out of 31). Among the 20 patients experiencing pain, 100% (*n* = 20) reported pain in the neck, 55% (*n* = 11) in the upper back, 50% (*n* = 10) in the head, 50% (*n* = 10) in the shoulder, and 25% (*n* = 5) in the arm. Sixteen of them (80%) used simple analgesics or NSAIDs, two (10%) used opioids and the remaining two (10%) did not use any oral pain medication. Table 1 shows the descriptive features of the entire sample.

The mean CSI score of the total sample was 38.3 (SD: 15.2), and 15 out of 31 (48.4%) patients had a CSI score ≥40 points, suggesting the presence of sensitization-associated symptoms (CSI score: 50.7, SD: 7.9). Most patients scoring ≥40 on the CSI (13/15, 86.6%) reported pain. The mean S-LANSS score for the 20 patients experiencing pain was 5.3 (SD: 5.5). Among these, five (25%) patients had a score ≥12 points, indicative of neuropathic-like symptoms, with a mean score of 14.6 (SD: 2.4). 

The mean HADS-A score for the entire sample was 8.6 (SD: 4.3), and 8 out of 31 (25.8%) patients had a score of ≥12 points, indicating potential anxiety (HADS-A score: 13.9, SD: 2.0). The mean HADS-D score of the total sample was 6.1 (SD: 3.6), and 6 out of 31 (19.4%) patients scored ≥10 points suggesting the presence of depressive symptoms (mean score: 10.3, SD: 0.5). Additionally, the mean PSQI score for the entire sample was 8.7 (SD: 4.0), with 18 out of 31 (58.1%) participants scoring ≥8 points, consistent with poor sleep quality (mean score: 11.5, SD: 2.5). 

### 3.1. Differences between Patients with and without Pain

When comparing patients experiencing pain (*n* = 20) and those without pain (*n* = 11), the former showed higher scores on the TWSTRS disability subscale and the TWSTRS total score, as well as higher scores on the CSI (all, *p* < 0.001). Patients with pain also exhibited lower PPTs at the C5-C6 joint (*p* = 0.014), the second interossei space (*p* = 0.016) and the tibialis anterior (*p* = 0.010) when compared with those without pain (Table 2). No significant differences were found between the two groups in the scores of the TWSTRS severity subscale, the HADS-A, the HADS-D, or the PSQI.

### 3.2. Bivariate Correlation Analysis

Table 3 shows the correlation matrix of the outcomes. The CSI score was positively associated with the TWSTRS subscales for pain (r = 0.545, *p* = 0.002) and disability (r = 0.557, *p* = 0.001), the total TWSTRS score (r = 0.567, *p* = 0.001), S-LANSS (r = 0.643, *p* < 0.001), HADS-A (r = 0.611, *p* < 0.001), HADS-D (r = 0.599, *p*< 0.001) and PSQI (r = 0.578, *p* = 0.001). Moreover, the CSI score was negatively associated with the PPTs at all the assessed points (−0.540 < r < −0.510, all *p* < 0.01). 

In addition to correlating with the CSI, the S-LANSS score was positively associated with the TWSTRS subscales for pain (r = 0.594, *p* < 0.001) and disability (r = 0.754, *p* < 0.001), the total TWSTRS total score (r = 0.682, *p* < 0.001), HADS-A (r = 0.469, *p* < 0.008) and HADS-D (r = 0.390, *p*= 0.03). Also, the S-LANSS was negatively associated with the PPTs at all the assessed points (−0.587 < r < −0.466, all *p* < 0.01).

### 3.3. Multiple Regression Analysis

Hierarchical regression analyses revealed that both HADS-D (contributing 58.8%) and S-LANSS (contributing an additional 13.7%) contributed to CSI score and, when combined, they accounted for 72.5% of the variance (r2 adjusted: 0.725, Table 4). In addition, the stepwise regression analyses revealed that CSI was the only variable contributing to S-LANSS, explaining 42.3% of its variance (r2 adjusted: 0.423, Table 4).

## 4. Discussion

Our data show that pain is prevalent in CD, reaching up to 64.5% of patients in our sample. Interestingly, the presence of pain was not associated with a more severe dystonic pattern deviation but did relate to higher levels of disability. In fact, pain and other non-motor symptoms are the main drivers of reduced quality of life in CD patients and the first reasons why patients seek medical care [6,27]. 

This is the first study investigating the presence of sensitization-associated and neuropathic-like features and their contributing factors in patients with CD. By using two patient-reported outcome measures, i.e., the CSI and the S-LANSS, we found that almost 50% of CD patients showed sensitization-associated symptoms and 25% of CD patients experiencing pain showed neuropathic-like features. Sensitization-associated symptoms were more frequent in CD patients with pain than in those without pain. Furthermore, an examination of pressure sensitivity across different areas revealed that CD patients with pain had lower PPTs compared to those without pain.

### 4.1. Sensitization-Associated Symptomatology in Cervical Dystonia

Patients with chronic pain are known to have altered nociceptive processing [10]. Correspondingly, current theories propose that this altered nociceptive processing could be involved in CD-related pain [9]. Nonetheless, few published studies have investigated this phenomenon in CD from a multidimensional perspective.

Generalized hypersensitivity to pressure pain (i.e., pressure hyperalgesia) is one of the manifestations of central sensitization in chronic pain conditions [28]. In a sample of nine CD patients, Lobbezoo et al. found decreased PPTs in the sternocleidomastoid, upper trapezius and masseter muscles compared to healthy subjects [13]. In another study, Paracka et al. observed mechanical allodynia with pinprick and tactile stimulation but not pressure pain hyperalgesia on the dorsum of the hand and shoulder of patients with CD; yet the number of patients was small (*n* = 7) [29]. Our study does not allow us to confirm that there is pressure pain hyperalgesia in patients with CD, since we did not include a control group. However, compared to patients without pain, patients with pain exhibited lower PPTs at the C5-C6 joint, second interossei space and tibialis anterior. These findings suggest that CD patients experiencing pain may have a widespread disruption in pain processing rather than just a localized process originating in the musculoskeletal areas affected by dystonic movements.

In our study, an additional approach was used to indirectly assess the presence of sensitization-associated symptomatology by incorporating the CSI. According to the CSI, almost 50% of our patients had symptoms related to central sensitization. Of note, patients in the pain group had higher scores on the CSI than those patients in the non-pain group. Moreover, CSI scores were negatively correlated with PPTs; as patients reported more symptoms associated with sensitization, there was an increase in their pain sensitivity to pressure. However, these associations were not identified in the regression model. In fact, depressive symptomatology (HADS-D) was the main predictor of CSI. This finding is consistent with previous research arguing that exclusive use of CSI to infer sensitization is not recommended because it overlaps with psychological constructs [30]. In any case, the presence of psychological disturbances such as depression or anxiety have been shown to be significant determinants of health-related quality of life in patients with dystonia [31]. Accordingly, routine examination of patients with CD should include evaluation of these non-motor symptoms for a comprehensive assessment.

Our results support the presence of sensitization in patients with CD. However, we cannot determine the cause of this process. Thus, central sensitization may be linked to long-lasting nociceptive stimuli leading to increased synaptic excitability of central nervous system circuits or to an impairment of descending inhibitory pain mechanisms [32]. It seems that ascending nociceptive pathways are preserved in patients with CD but there is an alteration in conditioned pain modulation mechanisms [33]. Further studies are needed to identify the underlying mechanisms behind the altered nociceptive processing in CD.

### 4.2. Neuropathic-like Features in Cervical Dystonia Related-Pain

To date, no study has explored the presence of neuropathic-like symptoms in patients with CD. In our study, we introduced the S-LANSS questionnaire to analyze whether CD-related pain could have characteristics of neuropathic pain. According to this questionnaire, in the group of CD patients experiencing pain, 25% exhibited neuropathic-like features. However, we should be cautious with this finding because neuropathic pain cannot be diagnosed from a self-reported questionnaire alone, as additional tests and quantitative measurements would be required.

We also observed that S-LANSS score correlated negatively with PPT levels and positively with the CSI. Furthermore, regression analysis showed that CSI score was a predictor of S-LANSS, and vice versa. Thus, neuropathic-like pain features appeared to be related to sensitization-associated symptomatology. It is now increasingly recognized that neuropathic features, e.g., hyperalgesia and allodynia, may be present in nociplastic pain syndromes without evidence of nerve damage, representing an overlap of symptoms in a “chronic pain continuum”. Therefore, neuropathic-like symptoms might not always imply a structural lesion but be part of nociplastic mechanisms [34]. These mechanisms could possibly lead to neuropathic-like features in some patients with CD.

### 4.3. Towards Comprehensive Phenotyping of Pain in Cervical Dystonia

Current results suggest that the evaluation of pain in CD should not be limited to musculoskeletal or nociceptive mechanisms. The presence of symptoms associated with sensitization, pressure sensitivity to pain, as well as other symptomatology such as mood disorders or sleep problems, would suggest that pain in CD may present nociplastic features [11,12].

Although nociplastic pain is well recognized in the current literature, its definition has raised some questions [35]. First, distinguishing between the different pain phenotypes (nociceptive, neuropathic, and nociplastic pain) can be challenging in clinical practice because patients may fit more than one phenotype, as one phenotype does not exclude others [36]. A second challenge is the proper clinical determination of altered nociceptive pain processing (sensitization) since no gold standard exists. The IASP proposed the first set of clinical criteria and a grading system for identifying among the different pain phenotypes [37]. These criteria have been considered comprehensive, robust, properly developed, and with a high potential to be applied by clinicians [38], but they have never been applied to patients with CD. 

The recognition of these different pain mechanisms in CD may have implications in clinical practice. Nociplastic and functional pain syndromes are being reframed as biopsychosocial conditions that benefit from multimodal and integrative treatments [12]. Advances in pharmacology and molecular mechanisms must be combined with tailored plans that include neuroscience education, cognition-targeted exercises, and management of factors such as mood, sleep, and overall well-being [39].

### 4.4. Limitations

This is the first study investigating the presence of sensitization and neuropathic-like symptoms in CD, but some limitations should be recognized. First, although the sample included in our study is larger than that of previous studies [13,29], it is limited to just one hospital and may be considered relatively small to identify firm conclusions. Future studies including large samples are now needed to confirm or refute these results. Second, the cross-sectional design does not allow us to determine the role of sensitization-associated or neuropathic-like symptomatology in CD prognosis. Third, the heterogeneity of the sample in terms of head deviation patterns did not allow for statistical comparison between the different categories. In fact, the risk of pain in CD may vary depending on the pattern of head deviation [40]. We do not know if the presence of sensitization mechanisms would be also different between these CD phenotypes. Fourth, although we excluded patients with evidence of nerve damage, we did not perform specific tests to rule out other causes of structural nervous lesions (e.g., small fiber neuropathy). Fifth, all participants were patients regularly treated with botulinum toxin injections, and many of them were taking oral pain medications. Although data collection was conducted at least three months after the last botulinum toxin injection and at least one day after the last analgesic medication intake, these medications could affect nociceptive processing in the long-term. Finally, we have used different questionnaires to assess different aspects of pain, but they are not adapted to CD. Bruno et al. recently developed the Pain in Dystonia Scale (PIDS), the first specific tool designed to assess pain and related disability in patients with CD [41]. The PIDS should therefore be used in future studies evaluating the functional consequences of pain in CD.

## 5. Conclusions

This study found that pain is a common non-motor symptom in patients with CD, which is associated with increased disability but not with the severity of dystonia. Almost half of patients with CD had sensitization-associated symptoms which were associated with higher pressure pain sensitivity. Among the CD patients experiencing pain, 25% exhibited neuropathic features despite the absence of evident damage in the peripheral or central nervous system. In conclusion, pain is an important source of disability in CD, and may be a consequence of different mechanisms. Identifying all these mechanisms may be crucial for a tailored and personalized treatment of pain and disability in CD. 

## Figures and Tables

**Table 1:** Descriptive characteristics of the sample (*n* = 31).

Demographic Variables	
Age (years), mean (SD)	61.2 (10.1)
Sex, *n* (%) Female Male	23 (74.2) 8 (25.8)
**Clinical variables related to dystonia and pain**	
Time since onset of CD (years), mean (SD)	15.4 (12.1)
Time since start of BT treatment (years), mean (SD) *	11.1 (9.5)
Head deviation, *n* (%) Torticollis Laterocollis Anterocollis Retrocollis	14 (45.2) 10 (32.3) 5 (16.1) 2 (6.5)
Head tremor, *n* (%)	23 (74.2)
Sensory tricks, *n* (%)	23 (74.2)
Patients with pain, *n* (%) Neck pain Upper back pain Head pain Shoulder pain Arm pain	20/31 (64.5) 20/20 (100) 11/20 (55) 10/20 (50) 10/20 (50) 5/20 (25)
Side of pain, *n* (%) Right Left Both	9/20 (45) 6/20 (30) 5/20 (25)
Intensity of pain (NPRS, 0–10), mean (SD)	6 (1.8)
Temporal pattern of pain, *n* (%) Continuous Intermittent	11/20 (55) 9/20 (45)
TWSTRS Severity score, mean (SD) Disability score, mean (SD) Pain score, mean (SD) Total score, mean (SD)	13.5 (5.1) 6.4 (6.3) 8 (6.9) 27.9 (13.4)
**Sensitization-associated and neuropathic-like symptoms**	
CSI (0–100), mean (SD)	38.3 (15.2)
S-LANSS (0–24), mean (SD)	5.3 (5.5)
**Psychological and sleep variables**	
HADS-A (0–21), mean (SD)	8.6 (4.3)
HADS-D (0–21), mean (SD)	6.1 (3.6)
PSQI (0–21), mean (SD)	8.7 (4.0)
**Pressure pain thresholds (Kg/cm^2^)**	
C5-C6 joint, mean (SD)	4.3 (2.1)
Upper trapezius, mean (SD)	3.1 (1.4)
Second interossei space, mean (SD)	3.9 (1.7)
Tibialis anterior, mean (SD)	6.1 (2.4)

* 17 patients were assessed at 3 months and 14 patients at 4 months after their last BT injection. BT: botulinum toxin; CD: cervical dystonia; CSI: Central Sensitization Inventory; HADS: Hospital Anxiety and Depression Scale (A: anxiety; D: depression); NPRS: Numerical Pain Rate Scale (0: no pain, 10: the worst pain imaginable); PSQI: Pittsburgh Sleep Quality Index; SD: standard deviation; S-LANSS: self-administered Leeds Assessment of Neuropathic Symptoms and Signs Scale; TWSTRS: Toronto Western Spasmodic Torticollis Rating Scale.

**Table 2:** Comparison between patients with cervical dystonia with and without pain.

Variables	Patients with Pain (*n* = 20)	Patients without Pain (*n* = 11)	*p*-Value
Age (years), mean (SD)	58.5 (9.5)	66.0 (9.0)	0.045
Sex (women/men), *n* (%)	13 (65)/7 (35)	10 (91)/1 (9)	0.045
Time with CD (years), mean (SD)	13.5 (10.0)	18.8 (15.0)	0.253
Head tremor, *n* (%)	15 (75)	8 (72.7)	0.686
Sensory tricks, *n* (%)	17 (85)	6 (54.5)	0.080
TWSTRS Severity score, mean (SD) Disability score, mean (SD) Pain score, mean (SD) Total score, mean (SD)	13.5 (5.6) 8.9 (6.3) 12.4 (4.1) 34.8 (11.3)	13.6 (4.3) 1.6 (2.6) ---- 15.3 (4.6)	0.944 <0.001 ---- <0.001
CSI (0–100), mean (SD)	42.9 (14.1)	30.0 (13.9)	<.001
S-LANSS (0–24), mean (SD)	8.3 (4.7)	----	----
HADS-A (0–21), mean (SD)	9.1 (4.4)	7.5 (4.0)	0.344
HADS-D (0–21), mean (SD)	6.7 (3.7)	5.1 (3.3)	0.257
PSQI (0–21), mean (SD)	8.9 (4.0)	8.4 (4.2)	0.662
PPTs (Kg/cm^2^) C5-C6 joint, mean (SD) Upper trapezius, mean (SD) 2nd interossei space, mean (SD) Tibialis anterior, mean (SD)	3.8 (2.2) 2.8 (1.4) 3.3 (1.7) 5.6 (2.7)	5.1 (1.7) 3.5 (1.2) 4.9 (1.4) 6.9 (1.7)	0.014 0.174 0.016 0.010

CD: cervical dystonia; CSI: Central Sensitization Inventory; HADS-A: Hospital Anxiety and Depression Scale (A: anxiety; D: depression); PPTs: Pressure Pain Thresholds; PSQI: Pittsburgh Sleep Quality Index; SD: standard deviation; S-LANSS: self-administered Leeds Assessment of Neuropathic Symptoms and Signs Scale; TWSTRS: Toronto Western Spasmodic Torticollis Rating Scale.

**Table 3:** Pearson-product moment correlation matrix between demographic, clinical and neurophysiological variables.

	1	2	3	4	5	6	7	8	9	10	11	12	13	14	15
**1.** Age															
**2.** Time with CD	n.s.														
**3.** Pain intensity (NPRS)	n.s.	n.s.													
**4.** TWSTRS severity	n.s.	0.472 ^#^	n.s.												
**5.** TWSTRS disability	n.s.	n.s.	n.s.	n.s.											
**6.** TWSTRS pain	n.s.	n.s.	0.681 ^#^	n.s.	0.590 ^#^										
**7.** TWSTRS total score	n.s.	n.s.	n.s.	0.485 ^#^	0.867 ^#^	0.788 ^#^									
**8.** CSI	n.s.	n.s.	n.s.	n.s.	0.545 ^#^	0.557 ^#^	0.567 ^#^								
**9.** S-LANSS	n.s.	n.s.	n.s.	n.s.	0.594 ^#^	0.754 ^#^	0.682 ^#^	0.643 ^#^							
**10.** HADS-A	n.s.	n.s.	n.s.	n.s.	0.440 ^#^	n.s.	n.s.	0.611 ^#^	0.469 ^#^						
**11.** HADS-D	n.s.	n.s.	n.s.	n.s.	0.565 ^#^	0.367 *	0.578 ^#^	0.599 ^#^	0.390 *	0.560 ^#^					
**12.** PSQI	n.s.	n.s.	n.s.	n.s.	0.435 ^#^	n.s.	−0.371 *	0.578 ^#^	n.s.	0.567 ^#^	0.517 ^#^				
**13.** C5-C6 joint PPT	n.s.	n.s.	n.s.	n.s.	n.s.	n.s.	n.s.	−0.540 ^#^	−0.466 ^#^	−0.528 ^#^	−0.507 ^#^	n.s.			
**14.** Upper trapezius PPT	n.s.	n.s.	n.s.	n.s.	n.s.	n.s.	n.s.	−0.515 ^#^	−0.477 ^#^	−0.577 ^#^	−0.468 ^#^	n.s.	0.927 ^#^		
**15.** Second interossei space PPT	n.s.	n.s.	n.s.	n.s.	n.s.	n.s.	n.s.	−0.522 ^#^	−0.587 ^#^	−0.488 ^#^	−0.364 *	n.s.	0.838 ^#^	0.906 ^#^	
**16.** Tibialis anterior PPT	n.s.	n.s.	n.s.	n.s.	n.s.	n.s.	n.s.	−0.510 ^#^	−0.537 ^#^	−0.415 *	−0.344 *	n.s.	0.798 ^#^	0.792 ^#^	0.853 ^#^

CD: cervical dystonia; CSI: Central Sensitization Inventory; HADS: Hospital Anxiety and Depression Scale (A: anxiety, D: depression); NPRS: Numerical Pain Rating Scale; PPT: Pressure Pain Threshold; PSQI: Pittsburgh Sleep Quality Index; S-LANSS: self-administered Leeds Assessment of Neuropathic Symptoms and Signs; TWSTRS: Toronto Western Torticollis Spasmodic Rating Scale. * *p* < 0.05; ^#^ *p* < 0.01; n.s.: non-significant.

**Table 4:** Summary of the stepwise regression analyses to determine predictors of sensitization-associated and neuropathic-like symptoms.

	Predictor Outcome	Β	SE B	95% CI	*B*	t	*p*
CSI	Step 1 (R^2^ adjusted = 0.588) HADS-D	2.936	0.554	1.773, 4.099	0.781	5.303	0.001
	Step 2 (R^2^ adjusted = 0.725) HADS-D S-LANSS	2.274 1.252	0.498 0.396	1.222, 3.325 0.416, 2.087	0.605 0.419	4.452 3.161	0.001 0.006
S-LANSS	Step 1 (R^2^ adjusted = 0.423) CSI	0.225	0.058	0.103, 0.348	0.673	3.863	0.001

CI: confidence interval: CSI: Central Sensitization Inventory; HADS: Hospital Anxiety and Depression Scale (D: Depression); S-LANSS: self-administered Leeds Assessment of Neuropathic Symptoms and Signs.

## Data Availability

The authors confirm that the data supporting the findings of this study are available within the article.
